# A rare case of pontine tegmental cap dysplasia

**DOI:** 10.1016/j.radcr.2024.12.042

**Published:** 2025-01-08

**Authors:** Mosab Maree, Islam Rajab, Andrew Edward Leung, Keng Yeow Tay, Shivaprakash B. Hiremath

**Affiliations:** a London Health Sciences Center, Western University, Department of Medical Imaging, London, Canada; b Faculty of Medicine and Health Sciences, An-Najah National University,Nablus, 44839, Palestine; c Department of Internal Medicine, St joseph University Medical Center, Paterson, NJ; d Department of Thoracic Radiology, Columbia University Irving Medical Center, New York, NY; e Division of Neuroradiology, Joint Department of Medical Imaging, Toronto Western Hospital, Department of Medical Imaging, University of Toronto, Toronto, Ontario

**Keywords:** PTCD, Genetics, Pontine Tegmental Cap Dysplasia

## Abstract

Pontine Tegmental Cap Dysplasia (PTCD) is a rare hindbrain malformation characterized by cranial nerve dysfunction, cerebellar abnormalities, and developmental delays of varying severity. This case report presents a 12-month-old female with significant developmental delays, hypotonia, and cranial nerve abnormalities. The findings underscore the critical role of radiology and neuroimaging in diagnosing and managing PTCD. Additionally, this case highlights the importance of neuroimaging in differentiating PTCD from other possible diagnoses and malformations

## Introduction

Pontine tegmental cap dysplasia (PTCD) is an uncommon neurological disorder characterized by a specific malformation of the hindbrain and dysfunction of the cranial nerves [1]. Clinically, patients exhibit hearing loss, multiple cranial nerve abnormalities, and cerebellar involvement, leading to symptoms such as hypotonia, ataxia, corneal opacity, feeding challenges, hearing impairment, and undeveloped speech. Some individuals also experience developmental delays, seizures, and pyramidal signs, which suggest potential involvement of supratentorial regions. Additionally, extracerebral features may include skeletal anomalies, congenital heart defects, and kidney malformations [2]. Here, we report the clinical and radiological findings in a case of PTCD, discussing the possible differential diagnoses.

## Case presentation

**Fig. 1:**
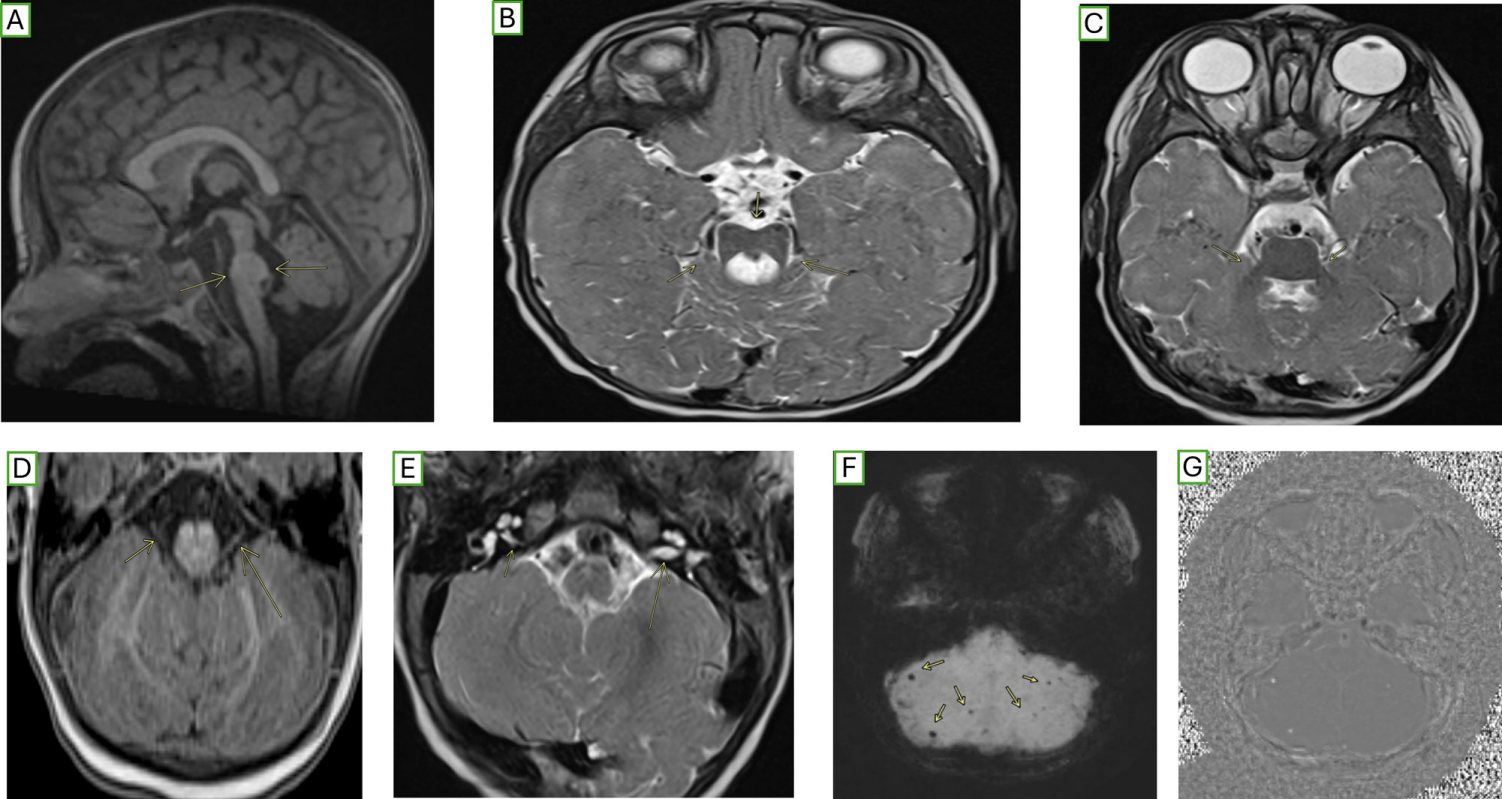
Midline sagittal T1-weighted imaging (A) demonstrates significant ventral flattening of the pons, with dorsal rounded fullness protruding into the fourth ventricle. Axial T2-weighted imaging at the level of the isthmus (B) reveals the classic “molar tooth” configuration, characterized by laterally displaced superior cerebellar peduncles and a deep interpeduncular fossa. Additional axial T2-weighted imaging (C) shows hypoplasia of the middle cerebellar peduncles. Axial T1-weighted imaging without contrast (D) depicts a smaller right cranial nerve VII/VIII complex; however, no high-resolution T2 images were available for further evaluation. Another axial T2-weighted image (E) highlights a smaller right internal auditory canal compared to the left. Finally, axial susceptibility-weighted (F) and phase (G) imaging demonstrate several foci of susceptibility artifact in both cerebellar hemispheres, consistent with prior microhemorrhages, without evidence of mass effect or edema.

The patient is a 12-month-old female with a complex medical history, including premature birth at 35 weeks, requiring a 4-week neonatal intensive care unit (NICU) stay for feeding and growth issues, and prenatal exposure to cannabis. She failed her newborn hearing screen, and no additional audiologic evaluation or follow-up was conducted. She presented to the primary care clinic with a persistent nonhealing wound on her lower lip, likely from an overheated formula burn. On physical examination, her temperature was 36.3°C (axillary), heart rate 112 beats per minute, respiratory rate 30 breaths per minute, blood pressure 84/70 mmHg, and oxygen saturation (SpO2) of 99%. Measurements revealed a length of 65 cm, weight of 7.6 kg (15th percentile), and head circumference of 42.4 cm (3rd to 10th percentile). The patient otherwise appeared well with no signs of distress. Cardiovascular examination revealed normal heart sounds with no murmurs. The head, eyes, ears, nose, and throat (HEENT) examination showed no dysmorphic features; however, a lip lesion of 1 cm x 1.5 cm showed full-thickness mucosal involvement and partial muscle penetration. A punch biopsy of the wound was performed due to the unclear etiology, with concern for possible tumor or infection. Pathology revealed a clean wound with healthy bleeding. Conservative management, including saline rinses post-feeding and Vaseline application, was recommended. Concurrent significant global developmental delay (GDD) prompted a comprehensive outpatient workup. Developmental delays were noted from around 3 months, including poor central tone, inability to sit unsupported, limited head control, a raking grasp without a pincer grasp, absence of babbling or words, and frequent coughing and choking during feeding. Further investigations included Magnetic Resonance Imaging (MRI), which revealed abnormal contours of the pons, abnormal superior and middle cerebellar peduncles, a hypoplastic right cranial nerve VII/VIII complex, and a small right internal auditory canal, all highly suggestive of pontine tegmental cap dysplasia (Fig. 1).

A genetics consultation was performed to explore potential genetic associations with PTCD, but no syndromic features were identified.

The patient underwent nasogastric (NG) tube insertion for aspiration protection, followed by gastrostomy tube (G-tube) placement without complications. An examination under anesthesia (EUA) of the mouth and ears was conducted, showing no abnormalities apart from the oral wound. Further neurological assessment revealed significantly reduced truncal tone, head lag, and mild dyspraxia. The diagnosis of PTCD was established, and the patient required close outpatient follow-up tailored to her condition.

## Discussion

Pontine tegmental cap dysplasia is a rare hindbrain malformation, with Apporximatly 50 patients reported till date in the literature [3].

Barth and colleagues introduced the term PTCD first in 2007 in their report of 4 cases [4]. However, 2 potential cases exhibiting similar hindbrain malformations and clinical features were documented before 2007 [5,6].

Embryologically, the structures of the posterior fossa originate from the hindbrain. The pons, cerebellum, and superior portion of the fourth ventricle develop from the metencephalon, while the medulla and inferior portion of the fourth ventricle arise from the myelencephalon. During the sixth week of gestation, the pontine flexure divides the fourth ventricle into anterior and posterior membranous areas, with the cerebellar vermis forming in the anterior membranous area. PTCD is a sporadic malformation with an unknown genotype and no familial recurrence, its pathogenesis remains unclear, but it is hypothesized to result from defective migration or navigation of axons of rhombencephalic neurons [7].

On MRI, PTCD findings reveal a hindbrain malformation as characterized by a flattened, hypoplastic ventral pons accompanied by a tegmental cap, which is a curved protrusion of the dorsal pons extending from the middle third of the tegmentum into the fourth ventricle [8].

Frequently, the middle or inferior cerebellar peduncles are absent or underdeveloped, which helps distinguish this condition from Moebius syndrome, which can present with similar symptoms [8,9]. Other imaging characteristics include a molar tooth appearance of the pontomesencephalic junction, absence of inferior olivary prominences, and variable absence of cranial nerves V, VI, VII, VIII, and IX [10].

All documented cases exhibit a wide spectrum of symptoms. While clinical findings are variable, the involvement of some cranial nerves is almost always observed. Cranial nerve VIII Impairment, leading to sensorineural deafness, is reported in all patients. Consequently, all patients experienced speech impairment, ranging from complete muteness and use of sign language to some level of recognizable speech. Three previously reported patients received cochlear implants, which improved speech in some cases, while one patient showed minimal response [2,11]. In our patient MRI revealed hypoplastic right cranial nerve VII/VIII, and a small right internal auditory canal.

PTCD can be distinguished from conditions such as global cerebellar hypoplasia (GCH), pontocerebellar hypoplasia (PCH), and Moebius syndrome. GCH features a reduced cerebellar volume while maintaining a nearly normal shape, often caused by chromosomal abnormalities, infections, metabolic disorders, and syndromes with multiple anomalies. It is diagnosed through imaging that shows cerebellar dimensions below the normal range. Patients typically present with developmental delay, ataxia, hypotonia, and movement disorders. PCH, a group of autosomal recessive neurodegenerative disorders, involves prenatal abnormal development of the cerebellum and pons and is classified into ten subtypes based on clinical and genetic features. All subtypes exhibit cerebellar and pontine hypoplasia or atrophy, microcephaly, and variable cerebral involvement. Unlike PTCD, PCH does not display ectopic dorsal pontine tissue on imaging, though it does show ventral pons flattening and reduced cerebellar dimensions [7,12,13].

Moebius syndrome is marked by bilateral VI and VII nerve palsies, which are not frequent in PTCD. Moebius syndrome also includes absent hypoglossal prominence, hypoplasia of the dorsal pons, and absence of the medial colliculus at the pontine level, leading to a depression in the fourth ventricle [14]. PTCD is frequently associated with developmental delays, oculomotor apraxia, ataxia, and sensorineural hearing loss (SNHL), which is almost universally present due to anomalies such as hypoplastic or absent vestibulocochlear nerves (VCNs) and duplicated internal auditory canals (IACs) [15]. In contrast, Möbius syndrome is predominantly characterized by feeding difficulties and visual impairments, while hearing loss being infrequent, affecting only 6.8% of cases [16].

PTCD shares a molar tooth sign with Joubert syndrome-related disorders (JSRD), caused by the absence of decussation of the superior cerebellar peduncles. However, in PTCD, the peduncles are less elongated and horizontal. A distinguishing feature of PTCD is the pontine tegmental cap, which is absent in Joubert syndrome. In contrast, Joubert syndrome is characterized by hypotonia, abnormal eye movements, retinopathy, nephronophthisis, hepatic fibrosis, and polydactyly [17].

The prognosis of PTCD is highly variable, ranging from mild cognitive delay to severe disability [12].

## Conclusion

Pontine Tegmental Cap Dysplasia (PTCD) is an extremely rare neurological disorder with a broad spectrum of clinical manifestations, including cranial nerve dysfunction and developmental delays. The case highlighted classic imaging findings characteristic of this syndrome.

## Ethical approval

Ethical approval is exempt/waived at our institution.

## Availability of data and materials

The dataset supporting the conclusions of this article is included within the article.

## Provenance and peer review

Not commissioned, externally peer-reviewed.

## Author contributions statement

MM and IR were major contributors in manuscript writing. The remaining authors contributed to draft revision and editing. All authors read and approved the final manuscript.

## Patient consent

A written informed consent was obtained for the publication of this case report and accompanying images. A copy of the written consent is available for review by the Editor-in-Chief of this journal upon request.
